# Identification and Characterization of MicroRNAs by High Through-Put Sequencing in Mesenchymal Stem Cells and Bone Tissue from Mice of Age-Related Osteoporosis

**DOI:** 10.1371/journal.pone.0071895

**Published:** 2013-08-21

**Authors:** Xiaoning He, Wenkai Zhang, Li Liao, Xin Fu, Qing Yu, Yan Jin

**Affiliations:** 1 Research and Development Center for Tissue Engineering, Fourth Military Medical University, Xi’an, Shaanxi, China; 2 Engineering technology center for tissue engineering of Xi’an, Shaanxi, China; 3 Department of Oral Histopathology, School of Stomatology, Fourth Military Medical University, Xi’an, Shaanxi, China; 4 Department of Endodontics, School of Stomatology, Fourth Military Medical University, Xi’an, Shaanxi, China; University of Sao Paulo - USP, Brazil

## Abstract

The functional deficiencies of bone marrow-derived mesenchymal stem cells (MSCs) may contribute to the aging process and age-related diseases, such as osteoporosis. Although it has been reported that microRNAs (miRNAs) played an important role in mechanisms of gene regulation of aging, and their expression profiles in MSCs osteogenic differentiation were established in recent years, but it is still elusive for the dynamic patterns of miRNAs in aging process. Importantly, the miRNAs in aged bone tissue had not been yet reported so far. Here, we combined high through-put sequencing with computational techniques to detect miRNAs dynamics in MSCs and bone tissue of age-related osteoporosis. Among the detected miRNAs, 59 identified miRNAs in MSCs and 159 in bone showed significantly differential expressions. And more importantly, there existed 8 up-regulated and 30 down-regulated miRNAs in both MSCs and bone during the aging process, with the majority having a trend of down-regulation. Furthermore, after target prediction and KEGG pathway analysis, we found that their targeted genes were significantly enriched in pathways in cancer, which are complex genetic networks, comprise of a number of age-related pathways. These results strongly suggest that these analyzed miRNAs may be negatively involved in age-related osteoporosis, given that most of them showed a decreased expression, which could lay a good foundation for further functional analysis of these miRNAs in age-related osteoporosis.

## Introduction

Aging increases the risk to develop several diseases, such as osteoporosis, which is regarded as highly age-related [1], featuring the bone loss and susceptibility to fragility fractures [2], [3]. Theoretically, as an attractive therapeutic candidates for several diseases and degenerative applications, mesenchymal stem cells (MSCs) have also been evaluated as a vital factor in the development of osteoporosis, since it keeps the ability of self-renewal and differentiation into multiple cell types, including osteocytes, chondrocytes, and adipocytes [4], [5], [6], [7], [8]. Indeed, recently, it has been reported that the functional deficiencies of MSCs could lead to the declining of bone integrity and function in the elderly [9], [10]. Moreover, other studies showed that MSCs were involved in continuous maintenance and repair of bone during aging [9], [11], [12]. Environmentally, the neighboring aged tissues should also be taken into account, for they could exert potential impact on MSCs, further causing the decrease in regenerative potential of bone [13], [14], [15].

Furthermore, epigenetically, microRNAs (miRNA) could influence many basic cellular and pathological processes by regulating gene expression post-transcriptionally through binding to complementary sequences in the 3′ untranslated region (3′ UTR) of target mRNAs [16]. And recent studies showed that miRNAs participated in regulation of aging and a variety of age-associated pathways [17]. For example, Inukai et al. [18] screened miRNA expression by using brains from mice of different ages and found that there was a global downward trend of miRNA expression during aging. Additionally, Mori et al. [19] observed an attenuated miRNA processing in adipose tissues during aging in mice, worm, and human, suggesting that it could be a conserved feature of aging. Hence, as for osteoporosis, being an age-related disease, it may be closely linked to miRNA expressions. However, to date, only limited types of tissues or organs have been analyzed in the mouse and human [15]. Yet little is known about the miRNA expression in the bone of mouse at different ages.

Here, in this study, to acquire a better understanding of potential contributions of miRNAs to age-related osteoporosis, we used high through-put deep sequencing to perform a comprehensive survey of miRNA expressions in MSCs and bone tissue based on a mouse model of osteoporosis. This study could provide reliable evidence for further uncovering the development mechanisms of osteoporosis in human during aging.

## Results

### Construction of a Small RNA Library [20] by Deep Sequencing

#### MSCs

In order to detect the miRNAs dynamics in age-related osteoporosis, a small RNA library of MSCs samples in three groups were obtained by deep sequencing. Overall, 14,565,370 clean reads were detected in young group (2 m), 13,834,317 clean reads were detected in adult group (8 m), and 13,001,553 clean reads were detected in old group (25 m). After alignment to the mouse genome (mm9), the results indicated that 11,843,150 (81.31%) reads in young MSCs, 11,194,206 (80.92%) reads in adult MSCs and 10,666,211(82.04%) reads in old MSCs were matched to mm9. The small RNAs were classified into different categories according to their biogenesis and annotation. Among the total reads, the ratio of noncoding RNA, including rRNA, tRNA, snRNA and snoRNA accounted for 1.99%, 1.3% and 1.03% in young, adult and old group, respectively (Table S1). The remaining small RNAs were retained for further analysis.

#### Bone tissue

Total RNA of the bone tissue samples of femurs and tibiae from three groups were extracted, subsequently, we used high through-put sequencing to identify miRNA profiles in bone. 14,979,697 clean reads, 14,597,780 and 10,899,301 clean reads were detected in young, adult and old group, respectively. But unlike the MSCs, there had a much lower genome-matched rate in three bone groups. The ratio was 54.75% (8,201,928), 68.63% (10,017,983) and 64.00% (6,975,893) in young, adult and old bone, respectively. At the same time, the rates of rRNA, tRNA, snRNA and snoRNA were increased in bone; they were 32.8%, 28.8% and 18.9% in young, adult and old group, respectively (Table S1).

### MiRNAs Profiles in Age-related Osteoporosis

To investigate miRNA expressions in age-related osteoporosis, genome-aligned reads correspond to known miRNAs were compared with miRBase database (miRBase18) to obtain the miRNA count. The total 1157 miRNAs were detected; however, only 587, 578, and 574 miRNAs were expressed in 2 m-MSCs, 8 m-MSCs and 25 m-MSCs, respectively (Table S2). For bone tissue samples, there were 676, 657 and 603 miRNAs expressions in 2 m-bone, 8 m-bone and 25 m-bone, respectively (Table S3).

### Differential Expressions of Known miRNA

**Figure 1:**
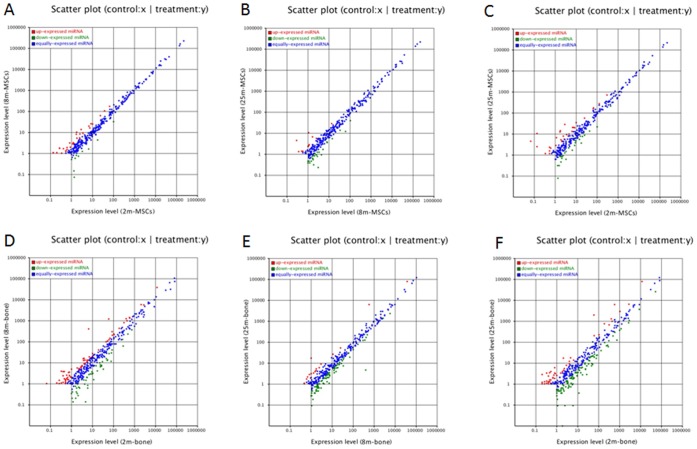
The scatter diagrams of miRNAs expressions between two samples. (**A**) 2m-MSCs versus 8m-MSCs. (**B**) 8m-MSCs versus 25m-MSCs. (**C**) 2m-MSCs versus 25m-MSCs. (**D**) 2m-bone versus 8m-bone. (**E**) 8m-bone versus 25m-bone. (**F**) 2m-bone versus 25m-bone.

**Figure 2:**
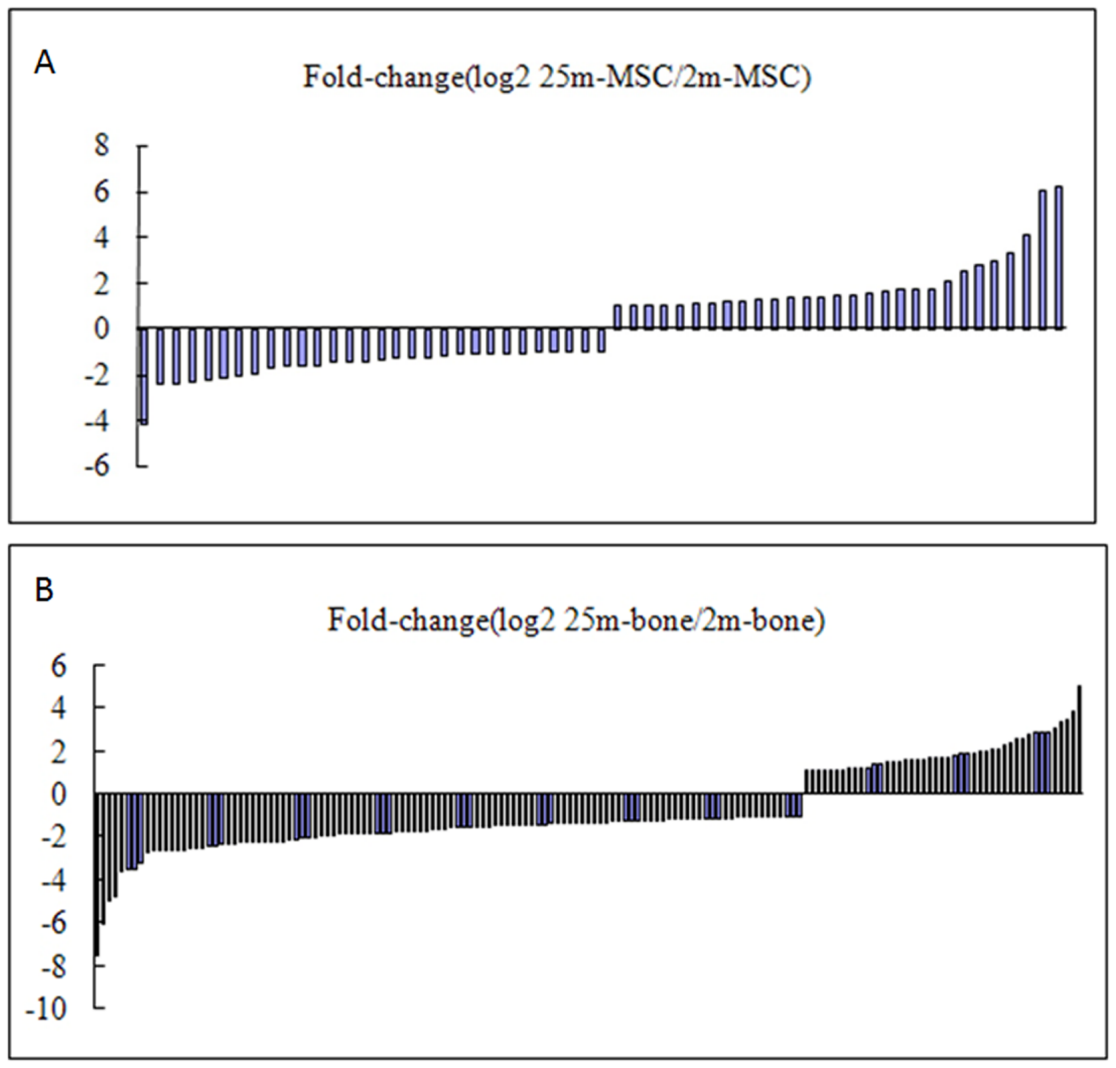
Expression changes of known miRNAs in age-related osteoporosis. Change more than 2.0-fold and P-value<0.05 (**A**) Distribution of individual miRNA expression changes in 2m and 25m MSCs. (**B**) Distribution of individual miRNA expression changes in 2m and 25m bone.

To analyze the variance of miRNAs that is differentially regulated during bone aging process of mice, we compared the known miRNA expressions between two samples to find out the differentially expressed miRNA. The expression of miRNA in two samples was shown by plotting Log2-ratio figure and scatter plot. For MSCs, the expression levels of miRNAs were shown in Table S4 and Figure 1A–C. For bone, the expression levels of miRNAs were shown in Table S5 and Figure 1D–F. To generate a convinced list of miRNAs variation in age-related osteoporosis, we filtered these data step by step. Firstly, selecting two-fold altered expression between the 2 m and 25 m samples, and ensuring P-value <0.05. The expression data were in Table S6. In MSCs, there were 59 miRNAs differently expressed, the miRNA expression changes were shown in Figure 2A. Comparing the numbers between down-regulated and up-regulated miRNAs (30 versus 29), we found that it reached a new homeostasis for the whole miRNA pattern in aged MSCs. In other words, there existed the transition of steady state of miRNA dynamics in different aged MSCs. However, from Figure 2B, we could find the changes of miRNA expressions in 2 m and 25 m bone, in 159 changed miRNAs, majority of them were down-regulated in old bone (114 versus 45). Interestingly, some miRNAs changes trends were opposite in MSCs and bone (red cells in Table S6). So, secondly, considering the miRNA expressions showed a consistent changing trend either decreasingly or increasingly among the three samples of MSCs and bone. Finally, screening the common miRNAs existed in the MSCs and bone, and ensuring the P- value <0.05 (Table S7). The results showed that there were 8 up-regulated and 30 down-regulated known miRNAs during bone aging (Table 1). Biological functions of corresponding miRNAs are involved in aging and osteoblastic differentiation was shown in Table 2.

**Table 1:** Summary of changed miRNAs during bone aging.

Up-regulated			Down-regulated		
miR-name	Fold change (Log2(25 m/2 m))		miR-name	Fold change (Log2(25 m/2 m))	
	MSCs	Bone		MSCs	Bone
miR-139-3p	1.1579667	1.0798472	let-7a-1-3p	−1.02878455	−0.67874004
miR-10b-5p	1.74235836	0.84854431	let-7c-2-3p	−1.02878455	−0.67874004
miR-1247-5p	2.57399688	0.85901003	miR-127-3p	−0.91365577	−2.24987567
miR-132-5p	0.78728182	1.91830046	miR-134-5p	−1.02048572	−4.75144550
miR-149-3p	2.50260213	1.09727177	miR-146a-5p	−0.96969902	−1.72036975
miR-1a-3p	1.21671956	2.71380958	miR-15a-5p	−0.81083050	−1.43912320
miR-223-5p	1.28061314	5.02582691	miR-16-5p	−0.67808853	−1.45915352
miR-532-5p	0.39765677	1.93682115	miR-1839-3p	−1.59247963	−1.11367240
			miR-210-3p	−0.34719066	−2.67051364
			miR-22-3p	−0.79861099	−1.48985084
			miR-296-5p	−0.50289888	−4.97584406
			miR-298-5p	−0.81414239	−2.64591680
			miR-299-5p	−1.37663478	−1.44810876
			miR-31-5p	−2.24764502	−0.66253663
			miR-329-3p	−2.42113547	−2.62866237
			miR-339-3p	−1.22850916	−1.06476259
			miR-362-3p	−0.46633041	−1.06669287
			miR-378-5p	−2.08390060	−1.80715166
			miR-379-5p	−1.26910522	−1.29609393
			miR-382-3p	−0.85660487	−2.20018664
			miR-382-5p	−1.41623538	−1.56871689
			miR-411-5p	−0.62727114	−2.62300693
			miR-433-3p	−0.65270266	−2.39918972
			miR-434-3p	−2.30943868	−1.92394154
			miR-467d-5p	−2.05850600	−2.20428267
			miR-485-5p	−1.05517307	−2.00064999
			miR-500-3p	−0.74720487	−1.45241652
			miR-574-3p	−0.79065800	−1.64790520
			miR-652-3p	−0.79357974	−1.29635004
			miR-674-3p	−0.49943749	−1.38044376

**Table 2:** Biological functions of corresponding miRNAs are involved in aging and osteoblastic differentiation.

miRNAs	targets	biological functions	Species
miR-10b-5p	unknown	Important regulatory factor in osteoblastic differentiation [21]	Mouse
miR-149-3p	Akt1 E2F1	Induced apoptosis [22]	human
miR-1a-3p	unknown	Upregulation in Zmpste24−/− mice modulating the levels of key componentsof the somatroph axis and DNA damage response pathways [23]	mouse
miR-223-5p	unknown	Increased in aged bone marrow derived dendritic cells [24]	human
miR-127-3p	Bcl6 Setd8	Suppressed cell growth [25]	rat
miR-146a-5p	IRAK1 TRAF6	Increased in aged bone marrow derived dendritic cells and compromisedBMDC function such as cytokine production during aging [24]	human
	NOX4	Important factor in regulating endothelial cell senescence [26]	
miR-15a-5p	unknown	Down-regulated in Ionizing radiation (IR) -induced senescence [27]	human
miR-16-5p	APP	Reduced amyloid protein precursor (APP) leads to a high risk of AD [28]	SAMP8 mouse
miR-210-3p	AcvR1b	Positive regulator of osteoblastic differentiation by inhibiting theTGF-beta/activin signaling pathway through inhibition of AcvR1b. [29]	ST2 stromal cells
	unknown	Induced double-strand DNA breaks and reactive oxygen speciesaccumulation in transfected cells [30]	
miR-22-3p	SIRT1 CDK6 SP1	Induced growth suppression and acquisition of a senescentphenotype in human normal and cancer cells [31]	human
	HDAC6	Promoted osteogenic differentiation and inhibits adipogenic differentiationof human adipose tissue-derived mesenchymal stem cells [32]	human
miR-31-5p	RhoBTB1	Repression of miR-31 inhibited colon cancer cell proliferation andcolony formation in soft agarose [33]	HT29 cells
	unknown	Is associated with marked change in the expression of specificmiRNA during aging in skeletal muscle [34]	mouse
miR-378-5p	NephronectinGalNT-7	Inhibited osteoblast differentiation [35]	MC3T3-E1
miR-382-5p	unknown	Downregulated in skeletal muscle of old mice [34]	mouse

**Figure 3:**
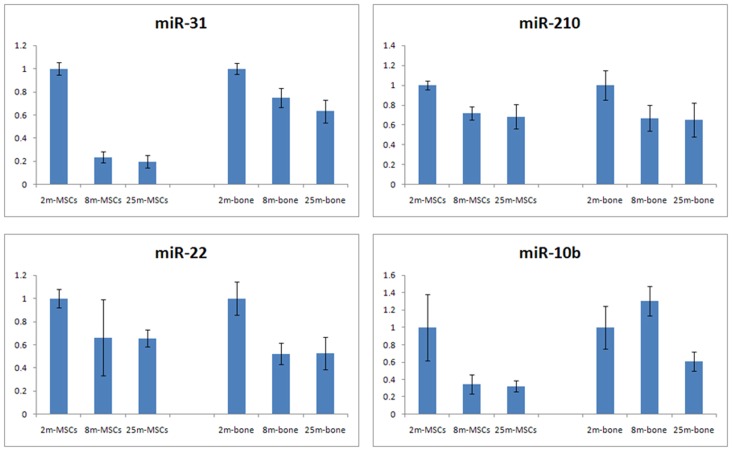
miRNA expression validated by qRT-PCR in MSCs and bone. qPCR results were normalized to U6 snRNA expression levels. Values showed that these miRNAs were significantly different between 2m and 25m samples.

In order to validate the sequencing data, we selected several miRNAs from Table 2 for additional qRT-PCR validation, which the minimum normalized read count of miRNAs was 5 in young, adult and old groups, including miR-210 [29], miR-22 [31], [32], miR-31 [36], [37], and miR-10b [16](Figure 3). Except for miR-10b, other miRNAs showed similar expression trends in qPCR and deep sequencing data. This result suggests that high through-put sequencing is an effective method for identifying mature miRNAs, and shows our prospect on the further research on the function of single miRNA.

### Computational Target Prediction of Changed miRNAs and Pathway Analysis

To identify the potential targets of changed miRNAs, we computationally identified mRNA targets of miRNAs using the software Mireap (software developed by BGI). To annotate the target genes corresponding to certain biological function during bone aging, we used GO (Gene Ontology) enrichment analysis to reveal the functions significantly related with predicted target gene candidates of changed miRNAs. The significantly enriched GO terms of biological process in genes repressed by changed miRNAs were shown in Table 3 and Table 4. To further understand the biological functions of changed miRNAs, KEGG pathway analysis was carried out among the predicted target genes of miRNAs. The KEGG analysis could reveal the main pathways which the target gene candidates are involved in. For up-regulated miRNAs, we found that cell cycle pathway (ko04110, P-value = 0.01621647), pathways in cancer (ko05200, P-value = 0.01038872) and calcium signaling pathway (ko04020, P-value = 0.006787428) were significantly targeted KEGG pathways (Figure S1A–C). These pathways were correlated with cell proliferation, osteogenic differentiation, and aging. And for down-regulated miRNAs, pathways in cancer (ko05200, P-value = 0.01555212) was significantly targeted KEGG pathway (Figure S1D).

**Table 3:** Functional annotation clusters of enriched GO biological processes predicted to be suppressed by up-regulated miRNAs in age-related osteoporosis.

GO term	biological processes	CorrectedP-Value
GO:0006810	transport	6.64e-06
GO:0045595	establishment of localization	1.44e-05
GO:0051234	regulation of metabolic process	0.00243
GO: 0048583	regulation of response to stimulus	0.00415
GO:0023051	regulation of signaling	0.00695
GO:0030154	cell differentiation	0.01235

**Table 4:** Functional annotation clusters of enriched GO biological processes predicted to be suppressed by down-regulated miRNAs in age-related osteoporosis.

GO term	biological processes	CorrectedP-Value
GO:0006810	transport	0.00036
GO:0051179	localization	0.02165
GO:0045595	establishment of localization	0.02944

### KEGG Pathways Analysis of Target Genes of miR-31

**Figure 4:**
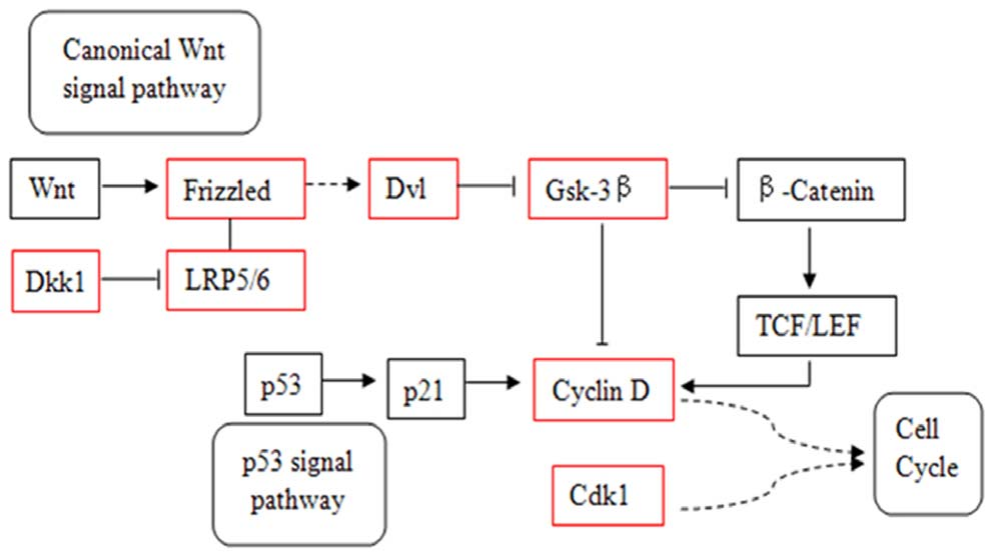
The correlated pathways were predicted to be targeted by miR-31. Red box: target genes of miR-31.

Due to the fact that each miRNA has hundreds of target genes, which function through multiple pathways, in either a positive or negative manner, it is rather difficult to perform a general evaluation of the effects of all the down-regulated miRNAs on aging and osteogenic differentiation in our study. Hence, here only miR-31 was chosen as representative, for it has been shown to be associated with aging and osteogenic differentiation in some other studies. After KEGG analysis, its target genes were indeed enriched in the p53 and Wnt signaling pathways, which are important parts of ko05200 (Figure 4, Table S8). Notably, based on the literature, p53 signaling pathway is associated with aging and Wnt pathway is associated with cell differentiation.

## Materials and Methods

### Animals

Male C57BL/6J mice (Experimental Animal Center of Fourth Military Medical University, China), 2 months-old (young group), 8 months-old (adult group) and 25 months-old (old group, osteoporosis) were used, each group had three samples.

### Ethics Statement

All animal experiments were performed under an animal study protocol approved by the ethics committee of Fourth Military Medical University.

### Cell Culture and Bone Collect

Femurs and tibiae were isolated from nine mice. The bone marrow was flushed out with α-MEM (Gibco, New York, NY, USA) containing 20% FBS (GIBCO, New York, NY, USA) and seeded in 90-mm dishes, cultures were refeeded every 3∼4 days, maintained about 15 days, then cells were passaged. The femurs and tibiae without bone marrow were stored in liquid nitrogen.

### RNA Preparation

Total cellular RNA was extracted from the second passage MSCs using Trizol Reagent (Invitrogen, Carlsbad, CA, USA), according to the manufacturer’s instructions. And the total RNA of femurs and tibiae was extracted as described by the office of shared research facilities in the University of Chicago (http://fgf.uchicago.edu/protocols_microarray.php), with slight modifications. In brief, guanidine buffer (containing 7.5 uLβ-ME per mL) was replaced by Trizol reagent, and phenol/chloroform/isoamyl alcohol was replaced by chloroform/isoamyl alcohol.

### High Through-put Deep Sequencing

High through-put deep sequencing was carried out using Hiseq2000 Sequencer (Illumina); the sequencing procedure was described by Chen et al. [38], [39]. Then the small RNA digitalization analysis based on the the image files generated by the sequencer were processed. The 50 nt sequence tags from HiSeq sequencing go through the data cleaning first, which included getting rid of the low quality tags and several kinds of contaminants from the 50 nt tags. Clean reads that correspond to known miRNAs were compared with a miRBase database (release 18.0) and the total copy number of each sample was normalized to 1,000,000. The data was processed using software developed by BGI. The high through-put sequencing data for this study have been submitted to the NCBI Sequence Read Archive (http://www.ncbi.nlm.nih.gov/Traces/sra) under accession number SRA072825 and SRA074415.

### Known MiRNA Expression Analysis

Considering the diversity of miRNA expression changes with age, we studied samples (MSCs and bone) from 2, 8 and 25 months-old mice to generate a convinced list of miRNAs variation in age-related osteoporosis. Primarily, we compared the known miRNA expressions between two samples, including 2 m versus 8 m, 8 m versus 25 m and 2 m versus 25 m. Then, the data were screened step by step. MiRNA expressions between the 2 m and 25 m samples were analyzed in detail, the miRNA with two-fold changed expressions (P-value<0.05) were selected. After that, we analyzed the data of 8 m samples, selected the miRNAs with consistent changing trend during aging process for further study. At last, we confirmed the convinced list of miRNAs variation by screening the common miRNAs existed in the MSCs and bone.

### MiRNA Target Prediction and Functional Analysis

Targets for known miRNAs were predicted using software Mireap (software developed by BGI). GO enrichment analysis of target gene candidates was carried out according to the GO terms in the database (http://www.geneontology.org/). KEGG pathway analysis is also used for the target gene candidates. Genes with FDR<0.05 were considered as significantly enriched in target gene candidates.

### Quantitative RT-PCR Assay

For real time PCR validation, miRNA cDNA was obtained according to specification of the One step PrimeScript miRNA cDNA synthesis kit (TaKaRa, Japan), Equal amounts of cDNA were used in duplicate and amplified with SYBR Premix Ex Taq II (TaKaRa, Japan). Mature DNA sense sequences (miRBase http://microrna.sanger.ac.uk/) of tested miRNAs were used as forward PCR primers. U6 snRNA was used as internal standard. All real time PCR reactions were performed on Bio-Rad CFX Manager. MiRNA fold changes between different groups were calculated by the 2^−ΔΔCt^ method. All assays were done with three wells per condition in three independent experiments.

### Statistics

Differences were statistically evaluated by Student’s t-test. Unless otherwise stated, P-value<0.05 was considered to be statistically significant.

## Discussion

Global expression profiling of miRNAs in various aged tissues or organs of different species have been performed. However, few data on miRNAs pattern are available so far in terms of age-related osteoporosis. In present results, we provided miRNAs dynamics in MSCs and bone tissue of age-related osteoporosis and further performed target prediction and KEGG pathway analysis, which strongly suggested that multiple miRNAs may be involved in this complex process.

As multipotent adult stem cells, MSCs are capable of differentiating into osteoblast cells, however, many studies proved that this ability declined with age [10], [12], [40], [41]. On the molecular level, an increasing number of miRNAs have been identified to regulate osteogenic differentiation of MSCs [8], [42], [43], [44]. Therefore, miRNAs are crucial in the bone aging process. However, aside from Wagner et al. [5] revealed an up-regulation of mir-371, mir-369-5p, mir-29c, mir-499 and let-7f upon in human MSCs replicative senescence, there was no reports for miRNA expression profiling of MSCs in aging process. In our results, compared with the young group, 30 down-regulated and 29 up-regulated miRNAs were identified in the old MSCs group. The overall level of changes was no significant difference. Due to the fact that one cell type failed to represent the overall miRNA alteration in one organism [15], so, next we compared the miRNAs expression changes between the young and old bone tissues. Among which in total 114 miRNAs and 45 miRNAs showed significant decreasing and increasing expressions, respectively. Notably, the majority of miRNAs were down-regulated (fold change ≥2) in bone, meaning that aged tissue environment might play important roles in bone aging process. After carefully screening step by step, 8 up-regulated and 30 down-regulated miRNAs in both MSCs and bone were confirmed. That is to say, during aging process, the bone exhibits predominant miRNA down-regulation, which was in agreement with other miRNA studies on aging tissues and organisms [16], [45]. However, compared with other studies, some known SA-mi-RNAs [46], like miR-34a [47], [48], miR-499 [5], [49], miR-605 [50], miR-17 [16], [51] and so on, there was no significant difference in our study. One possibility was the difference of tissue specific, or because of in our study only common miRNAs in MSCs and bone were considered.

Possibly, the descend trend could be associated with Dicer, since recently study has provided evidence that the Dicer was declined with age [17], this might explain the decreases of multiple miRNAs in aging process. Additionally, other groups demonstrated that Dicer deficiency in osteoblast dominantly suppressed the bone formation [52], [53], and further showed that Dicer generated miRNAs are essential for the bone formation. Therefore, it is speculated that the down-regulated miRNAs may play protective roles in the bone aging.

Large numbers of studies have reported that miRNAs regulate osteoblast differentiation and bone formation [42], [54]. So, the next interesting point for us is how these changed miRNAs functioned in the bone aging process. When referring to literature, the mechanism of miRNAs worked by inhibiting gene expression, including positive and negative transcriptional factor and cytokine regulators, which are target genes of miRNAs [55], and regulate bone phenotype through certain biological processes [54]. We firstly used GO enrichment analysis to reveal the biological processes related with predicted target gene candidates of changed miRNAs. Results showed that the GO term transport (GO:0006810) significantly was enriched in the predicted target genes of both up-regulated and down-regulated miRNAs. Especially for the up-regulated miRNA related target genes, they enriched in GO terms regulation of response to stimulus (GO: 0048583) and cell differentiation (GO: 0030154). This result suggests us the up-regulated miRNAs might suppress some genes associated with the ability to respond on external stimulus. For example, the response of the cells to higher ROS (reactive oxygen species) levels, the MSCs from old mice might reduce ROS defense for the low expression level of related genes, further resulting in bone aging [13]. On the other hand, osteogenic differentiation of MSCs decline with age is clear, so, the differentiation-related gene was inhibited by the up-regulated miRNAs might be one of the reasons that caused age-related osteoporosis. For example, miR-214, which displayed up-regulation, could target ATF4, a positive transcription factor regulated osteoblast function, to inhibit bone formation [56].

Some studies reported that miRNA can affect pathways involved in aging, including p53 pathway, IGF pathway, mTOR pathway and sirtuins [15]. In our study, we analyzed the KEGG pathways of target genes that were related with significant alterations in miRNA expressions. Cell cycle pathway, calcium signaling pathway and pathways in cancer were significantly targeted KEGG pathways of up-regulated miRNAs, thereinto, pathways in cancer were affect by down-regulated miRNAs as well. These results suggest that the up-regulated miRNAs inhibited their target genes, which might mediate cell cycle control and regulate calcium ion homeostasis, which were important factors influencing the MSCs aging and mineralisation. Moreover, cancer is an age-related disease as well, so the miRNA regulates the pathways in cancer was reasonable in our study. However, according to the Figure S1, almost all of genes in cancer pathways (ko05200) were targeted by up- and down-regulated miRNAs, especially for most down-regulated miRNAs, it was hard for us to make appropriate judgments on which pathways play more important roles during bone aging process. So, in this study, we selected one of validated miRNAs to study its enriched pathways of likely targets, and analyzed the genes with known roles in aging or osteogenesis.

MiR-31, which was down-regulated in our study, and some published studies reported the function of miR-31 in aging, radio-resistance, cancer and cell differentiation. For example, Lynam-Lennon et al [57] found that miR-31 directly targeted 13 genes involved in DNA repair to decrease cellular defense against DNA damage. Goff et al [58] reported that miR-31 might be an inhibitor of the osteocyte differentiation pathway. Another study also showed that miR-31 was under-expressed in osteo-differentiated MSCs [43]. Rokah et al [59] found that miR-31 regulated cell cycle related genes, like cyclinD1. In our result, miR-31 was down-regulated during bone aging process, after KEGG enrichment analysis, we found that several negative regulator of Wnt signal pathway, such as DKK1, GSK-3β and CCND1 were targeted by miR-31. It has been recognized that Wnt signaling plays an important role in the regulation of bone formation [60] and aging [61]. Moreover, we also found cyclinD1 was a target gene of miR-31, which was a crosstalk of Wnt pathway and p53 pathway. It is noteworthy that p53 is well known as an age-related protein [62], and has ability to decrease bone mass [63]. As stated above, miR-31 might play an important role in regulating the age-related osteoporosis through its target genes, which were regulators in Wnt and/or p53 signal pathways.

In conclusion, age-related osteoporosis is a complex process, so, it was impossible to specify individual miRNA or signal pathway has decisive influence on bone aging. It was important to know how the networks of miRNAs and signal pathways operate temporally; the research on the networks and roles of miRNAs and/or their targeting genes in age-related osteoporosis was our further object.

## Supporting Information

Figure S1 **Genes and KEGG pathways that are predicted to be targeted by changed miRNAs. (A)** Genes and cell cycle pathway targeted by up-regulated miRNAs. **(B)** Genes and pathways in cancer targeted by up-regulated miRNAs. **(C)** Genes and calcium signaling pathway targeted by up-regulated miRNAs. **(D)** Genes and pathways in cancer targeted by down-regulated miRNAs. Genes in red boxes indicate that are targeted by at least one miRNA.
(TIF)

Table S1 **The categories of MSCs and bone small RNAs in mice at different ages.**
(XLSX)

Table S2 **All known miRNAs expressed in MSCs from mice at different ages.**
(XLSX)

Table S3 **All known miRNAs expressed in bone from mice at different ages.**
(XLSX)

Table S4 **Comparison of known miRNAs expression levels in MSCs between two samples determined by deep sequencing.** *: greater than 2.0-fold changes and P-value<0.05; **: greater than 2.0-fold changes and P-value<0.01.
(XLSX)

Table S5 **Comparison of known miRNAs expression levels in bone between two samples determined by deep sequencing.** *: greater than 2.0-fold changes and P-value<0.05; **: greater than 2.0-fold changes and P-value<0.01.
(XLSX)

Table S6 **Two-fold altered expression between the 2m and 25m samples.**
(XLSX)

Table S7 **The common miRNAs existed in the MSCs and bone.** Green: down-regulated miRNAs in bone aging process; red: up-regulated miRNAs.
(XLSX)

Table S8 **Genes and pathways targeted by miR-31.**
(XLSX)

## References

[1] Pietschmann P, Rauner M, Sipos W, Kerschan-Schindl K (2009) Osteoporosis: an age-related and gender-specific disease–a mini-review. Gerontology 55: 3–12. 18948685 10.1159/000166209

[2] Sambrook P, Cooper C (2006) Osteoporosis. Lancet 367: 2010–2018. 16782492 10.1016/S0140-6736(06)68891-0

[3] Cummings SR, Melton LJ (2002) Epidemiology and outcomes of osteoporotic fractures. Lancet 359: 1761–1767. 12049882 10.1016/S0140-6736(02)08657-9

[4] Wagner W, Ho AD, Zenke M (2010) Different facets of aging in human mesenchymal stem cells. Tissue Eng Part B Rev 16: 445–453. 20196648 10.1089/ten.TEB.2009.0825

[5] Wagner W, Horn P, Castoldi M, Diehlmann A, Bork S, et al (2008) Replicative senescence of mesenchymal stem cells: a continuous and organized process. PLoS One 3: e2213. 18493317 10.1371/journal.pone.0002213 PMC2374903

[6] Jiang Y, Jahagirdar BN, Reinhardt RL, Schwartz RE, Keene CD, et al (2002) Pluripotency of mesenchymal stem cells derived from adult marrow. Nature 418: 41–49. 12077603 10.1038/nature00870

[7] Marion NW, Mao JJ (2006) Mesenchymal stem cells and tissue engineering. Methods Enzymol 420: 339–361. 17161705 10.1016/S0076-6879(06)20016-8 PMC4035029

[8] Lakshmipathy U, Hart RP (2008) Concise review: MicroRNA expression in multipotent mesenchymal stromal cells. Stem Cells 26: 356–363. 17991914 10.1634/stemcells.2007-0625 PMC2673465

[9] Rossi DJ, Jamieson CH, Weissman IL (2008) Stems cells and the pathways to aging and cancer. Cell 132: 681–696. 18295583 10.1016/j.cell.2008.01.036

[10] Stolzing A, Jones E, McGonagle D, Scutt A (2008) Age-related changes in human bone marrow-derived mesenchymal stem cells: consequences for cell therapies. Mech Ageing Dev 129: 163–173. 18241911 10.1016/j.mad.2007.12.002

[11] Abdallah BM, Haack-Sorensen M, Fink T, Kassem M (2006) Inhibition of osteoblast differentiation but not adipocyte differentiation of mesenchymal stem cells by sera obtained from aged females. Bone 39: 181–188. 16530029 10.1016/j.bone.2005.12.082

[12] Fehrer C, Lepperdinger G (2005) Mesenchymal stem cell aging. Exp Gerontol 40: 926–930. 16125890 10.1016/j.exger.2005.07.006

[13] Rando TA (2006) Stem cells, ageing and the quest for immortality. Nature 441: 1080–1086. 16810243 10.1038/nature04958

[14] Sethe S, Scutt A, Stolzing A (2006) Aging of mesenchymal stem cells. Ageing Res Rev 5: 91–116. 16310414 10.1016/j.arr.2005.10.001

[15] Kasper G, Mao L, Geissler S, Draycheva A, Trippens J, et al (2009) Insights into mesenchymal stem cell aging: involvement of antioxidant defense and actin cytoskeleton. Stem Cells 27: 1288–1297. 19492299 10.1002/stem.49

[16] Dhahbi JM, Atamna H, Boffelli D, Magis W, Spindler SR, et al (2011) Deep sequencing reveals novel microRNAs and regulation of microRNA expression during cell senescence. PLoS One 6: e20509. 21637828 10.1371/journal.pone.0020509 PMC3102725

[17] Chen LH, Chiou GY, Chen YW, Li HY, Chiou SH (2010) MicroRNA and aging: a novel modulator in regulating the aging network. Ageing Res Rev 9 Suppl 1S59–66. 20708718 10.1016/j.arr.2010.08.002

[18] Inukai S, de Lencastre A, Turner M, Slack F (2012) Novel microRNAs differentially expressed during aging in the mouse brain. PLoS One 7: e40028. 22844398 10.1371/journal.pone.0040028 PMC3402511

[19] Mori MA, Raghavan P, Thomou T, Boucher J, Robida-Stubbs S, et al (2012) Role of MicroRNA Processing in Adipose Tissue in Stress Defense and Longevity. Cell Metab 16: 336–347. 22958919 10.1016/j.cmet.2012.07.017 PMC3461823

[20] (2003) Prevention and management of osteoporosis. World Health Organ Tech Rep Ser 921: 1–164, back cover. 15293701

[21] Okamoto H, Matsumi Y, Hoshikawa Y, Takubo K, Ryoke K, et al (2012) Involvement of microRNAs in regulation of osteoblastic differentiation in mouse induced pluripotent stem cells. PLoS One 7: e43800. 22937097 10.1371/journal.pone.0043800 PMC3427148

[22] Lin RJ, Lin YC, Yu AL (2010) miR-149* induces apoptosis by inhibiting Akt1 and E2F1 in human cancer cells. Mol Carcinog 49: 719–727. 20623644 10.1002/mc.20647

[23] Ugalde AP, Espanol Y, Lopez-Otin C (2011) Micromanaging aging with miRNAs: new messages from the nuclear envelope. Nucleus 2: 549–555. 22064465 10.4161/nucl.2.6.17986

[24] Park S, Kang S, Min KH, Woo Hwang K, Min H (2012) Age-Associated Changes in MicroRNA Expression in Bone Marrow Derived Dendritic Cells. Immunol Invest. 10.3109/08820139.2012.717328 23252865

[25] Pan C, Chen H, Wang L, Yang S, Fu H, et al (2012) Down-regulation of MiR-127 facilitates hepatocyte proliferation during rat liver regeneration. PLoS One 7: e39151. 22720056 10.1371/journal.pone.0039151 PMC3376093

[26] Vasa-Nicotera M, Chen H, Tucci P, Yang AL, Saintigny G, et al (2011) miR-146a is modulated in human endothelial cell with aging. Atherosclerosis 217: 326–330. 21511256 10.1016/j.atherosclerosis.2011.03.034

[27] Wang Y, Scheiber MN, Neumann C, Calin GA, Zhou D (2011) MicroRNA regulation of ionizing radiation-induced premature senescence. Int J Radiat Oncol Biol Phys 81: 839–848. 21093163 10.1016/j.ijrobp.2010.09.048 PMC3056910

[28] Liu W, Liu C, Zhu J, Shu P, Yin B, et al (2012) MicroRNA-16 targets amyloid precursor protein to potentially modulate Alzheimer’s-associated pathogenesis in SAMP8 mice. Neurobiol Aging 33: 522–534. 20619502 10.1016/j.neurobiolaging.2010.04.034

[29] Mizuno Y, Tokuzawa Y, Ninomiya Y, Yagi K, Yatsuka-Kanesaki Y, et al (2009) miR-210 promotes osteoblastic differentiation through inhibition of AcvR1b. FEBS Lett 583: 2263–2268. 19520079 10.1016/j.febslet.2009.06.006

[30] Faraonio R, Salerno P, Passaro F, Sedia C, Iaccio A, et al (2012) A set of miRNAs participates in the cellular senescence program in human diploid fibroblasts. Cell Death Differ 19: 713–721. 22052189 10.1038/cdd.2011.143 PMC3307984

[31] Xu D, Takeshita F, Hino Y, Fukunaga S, Kudo Y, et al (2011) miR-22 represses cancer progression by inducing cellular senescence. J Cell Biol 193: 409–424. 21502362 10.1083/jcb.201010100 PMC3080260

[32] Huang S, Wang S, Bian C, Yang Z, Zhou H, et al (2012) Upregulation of miR-22 promotes osteogenic differentiation and inhibits adipogenic differentiation of human adipose tissue-derived mesenchymal stem cells by repressing HDAC6 protein expression. Stem Cells Dev 21: 2531–2540. 22375943 10.1089/scd.2012.0014 PMC3424982

[33] Xu RS, Wu XD, Zhang SQ, Li CF, Yang L, et al (2013) The tumor suppressor gene RhoBTB1 is a novel target of miR-31 in human colon cancer. Int J Oncol 42: 676–682. 23258531 10.3892/ijo.2012.1746

[34] Hamrick MW, Herberg S, Arounleut P, He HZ, Shiver A, et al (2010) The adipokine leptin increases skeletal muscle mass and significantly alters skeletal muscle miRNA expression profile in aged mice. Biochem Biophys Res Commun 400: 379–383. 20800581 10.1016/j.bbrc.2010.08.079 PMC3740337

[35] Kahai S, Lee SC, Lee DY, Yang J, Li M, et al (2009) MicroRNA miR-378 regulates nephronectin expression modulating osteoblast differentiation by targeting GalNT-7. PLoS One 4: e7535. 19844573 10.1371/journal.pone.0007535 PMC2760121

[36] Sun F, Wang J, Pan Q, Yu Y, Zhang Y, et al (2009) Characterization of function and regulation of miR-24-1 and miR-31. Biochem Biophys Res Commun 380: 660–665. 19285018 10.1016/j.bbrc.2009.01.161

[37] Guo L, Zhao RC, Wu Y (2011) The role of microRNAs in self-renewal and differentiation of mesenchymal stem cells. Exp Hematol 39: 608–616. 21288479 10.1016/j.exphem.2011.01.011

[38] Chen X, Li Q, Wang J, Guo X, Jiang X, et al (2009) Identification and characterization of novel amphioxus microRNAs by Solexa sequencing. Genome Biol 10: R78. 19615057 10.1186/gb-2009-10-7-r78 PMC2728532

[39] Chen X, Ba Y, Ma L, Cai X, Yin Y, et al (2008) Characterization of microRNAs in serum: a novel class of biomarkers for diagnosis of cancer and other diseases. Cell Res 18: 997–1006. 18766170 10.1038/cr.2008.282

[40] Roura S, Farre J, Soler-Botija C, Llach A, Hove-Madsen L, et al (2006) Effect of aging on the pluripotential capacity of human CD105+ mesenchymal stem cells. Eur J Heart Fail 8: 555–563. 16507351 10.1016/j.ejheart.2005.11.006

[41] Wilson A, Shehadeh LA, Yu H, Webster KA (2010) Age-related molecular genetic changes of murine bone marrow mesenchymal stem cells. BMC Genomics 11: 229. 20374652 10.1186/1471-2164-11-229 PMC2873471

[42] Taipaleenmaki H, Bjerre Hokland L, Chen L, Kauppinen S, Kassem M (2012) Mechanisms in endocrinology: micro-RNAs: targets for enhancing osteoblast differentiation and bone formation. Eur J Endocrinol 166: 359–371. 22084154 10.1530/EJE-11-0646

[43] Gao J, Yang T, Han J, Yan K, Qiu X, et al (2011) MicroRNA expression during osteogenic differentiation of human multipotent mesenchymal stromal cells from bone marrow. J Cell Biochem 112: 1844–1856. 21416501 10.1002/jcb.23106

[44] Schoolmeesters A, Eklund T, Leake D, Vermeulen A, Smith Q, et al (2009) Functional profiling reveals critical role for miRNA in differentiation of human mesenchymal stem cells. PLoS One 4: e5605. 19440384 10.1371/journal.pone.0005605 PMC2680014

[45] Izzotti A, Calin GA, Steele VE, Croce CM, De Flora S (2009) Relationships of microRNA expression in mouse lung with age and exposure to cigarette smoke and light. FASEB J 23: 3243–3250. 19465468 10.1096/fj.09-135251 PMC2735372

[46] Lafferty-Whyte K, Cairney CJ, Jamieson NB, Oien KA, Keith WN (2009) Pathway analysis of senescence-associated miRNA targets reveals common processes to different senescence induction mechanisms. Biochim Biophys Acta 1792: 341–352. 19419692 10.1016/j.bbadis.2009.02.003

[47] He L, He X, Lim LP, de Stanchina E, Xuan Z, et al (2007) A microRNA component of the p53 tumour suppressor network. Nature 447: 1130–1134. 17554337 10.1038/nature05939 PMC4590999

[48] Tazawa H, Tsuchiya N, Izumiya M, Nakagama H (2007) Tumor-suppressive miR-34a induces senescence-like growth arrest through modulation of the E2F pathway in human colon cancer cells. Proc Natl Acad Sci U S A 104: 15472–15477. 17875987 10.1073/pnas.0707351104 PMC2000550

[49] Yang X, Feng M, Jiang X, Wu Z, Li Z, et al (2009) miR-449a and miR-449b are direct transcriptional targets of E2F1 and negatively regulate pRb-E2F1 activity through a feedback loop by targeting CDK6 and CDC25A. Genes Dev 23: 2388–2393. 19833767 10.1101/gad.1819009 PMC2764491

[50] Xiao J, Lin H, Luo X, Wang Z (2011) miR-605 joins p53 network to form a p53:miR-605:Mdm2 positive feedback loop in response to stress. EMBO J 30: 524–532. 21217645 10.1038/emboj.2010.347 PMC3034018

[51] Hackl M, Brunner S, Fortschegger K, Schreiner C, Micutkova L, et al (2010) miR-17, miR-19b, miR-20a, and miR-106a are down-regulated in human aging. Aging Cell 9: 291–296. 20089119 10.1111/j.1474-9726.2010.00549.x PMC2848978

[52] Mizoguchi F, Izu Y, Hayata T, Hemmi H, Nakashima K, et al (2010) Osteoclast-specific Dicer gene deficiency suppresses osteoclastic bone resorption. J Cell Biochem 109: 866–875. 20039311 10.1002/jcb.22228

[53] Gaur T, Hussain S, Mudhasani R, Parulkar I, Colby JL, et al (2010) Dicer inactivation in osteoprogenitor cells compromises fetal survival and bone formation, while excision in differentiated osteoblasts increases bone mass in the adult mouse. Dev Biol 340: 10–21. 20079730 10.1016/j.ydbio.2010.01.008 PMC2840721

[54] Lian JB, Stein GS, van Wijnen AJ, Stein JL, Hassan MQ, et al (2012) MicroRNA control of bone formation and homeostasis. Nat Rev Endocrinol 8: 212–227. 22290358 10.1038/nrendo.2011.234 PMC3589914

[55] Filipowicz W, Bhattacharyya SN, Sonenberg N (2008) Mechanisms of post-transcriptional regulation by microRNAs: are the answers in sight? Nat Rev Genet 9: 102–114. 18197166 10.1038/nrg2290

[56] Wang X, Guo B, Li Q, Peng J, Yang Z, et al (2013) miR-214 targets ATF4 to inhibit bone formation. Nat Med 19: 93–100. 23223004 10.1038/nm.3026

[57] Lynam-Lennon N, Reynolds JV, Marignol L, Sheils OM, Pidgeon GP, et al.. (2012) MicroRNA-31 modulates tumour sensitivity to radiation in oesophageal adenocarcinoma. J Mol Med (Berl). 10.1007/s00109-012-0924-x 22706599

[58] Goff LA, Boucher S, Ricupero CL, Fenstermacher S, Swerdel M, et al (2008) Differentiating human multipotent mesenchymal stromal cells regulate microRNAs: prediction of microRNA regulation by PDGF during osteogenesis. Exp Hematol 36: 1354–1369. 18657893 10.1016/j.exphem.2008.05.004 PMC2782644

[59] Rokah OH, Granot G, Ovcharenko A, Modai S, Pasmanik-Chor M, et al (2012) Downregulation of miR-31, miR-155, and miR-564 in chronic myeloid leukemia cells. PLoS One 7: e35501. 22511990 10.1371/journal.pone.0035501 PMC3325224

[60] Hay E, Laplantine E, Geoffroy V, Frain M, Kohler T, et al (2009) N-cadherin interacts with axin and LRP5 to negatively regulate Wnt/beta-catenin signaling, osteoblast function, and bone formation. Mol Cell Biol 29: 953–964. 19075000 10.1128/MCB.00349-08 PMC2643807

[61] DeCarolis NA, Wharton KA Jr, Eisch AJ (2008) Which way does the Wnt blow? Exploring the duality of canonical Wnt signaling on cellular aging. Bioessays 30: 102–106. 18200563 10.1002/bies.20709

[62] Levine AJ, Oren M (2009) The first 30 years of p53: growing ever more complex. Nat Rev Cancer 9: 749–758. 19776744 10.1038/nrc2723 PMC2771725

[63] Wang X, Kua HY, Hu Y, Guo K, Zeng Q, et al (2006) p53 functions as a negative regulator of osteoblastogenesis, osteoblast-dependent osteoclastogenesis, and bone remodeling. J Cell Biol 172: 115–125. 16380437 10.1083/jcb.200507106 PMC2063539

