# Supplementary figures and images for: Identification and Characterization of MicroRNAs by High Through-Put Sequencing in Mesenchymal Stem Cells and Bone Tissue from Mice of Age-Related Osteoporosis
Source: PLoS One. 2013 Aug 21;8(8):e71895. doi: 10.1371/journal.pone.0071895 (PMC3749187; doi:10.1371/journal.pone.0071895)

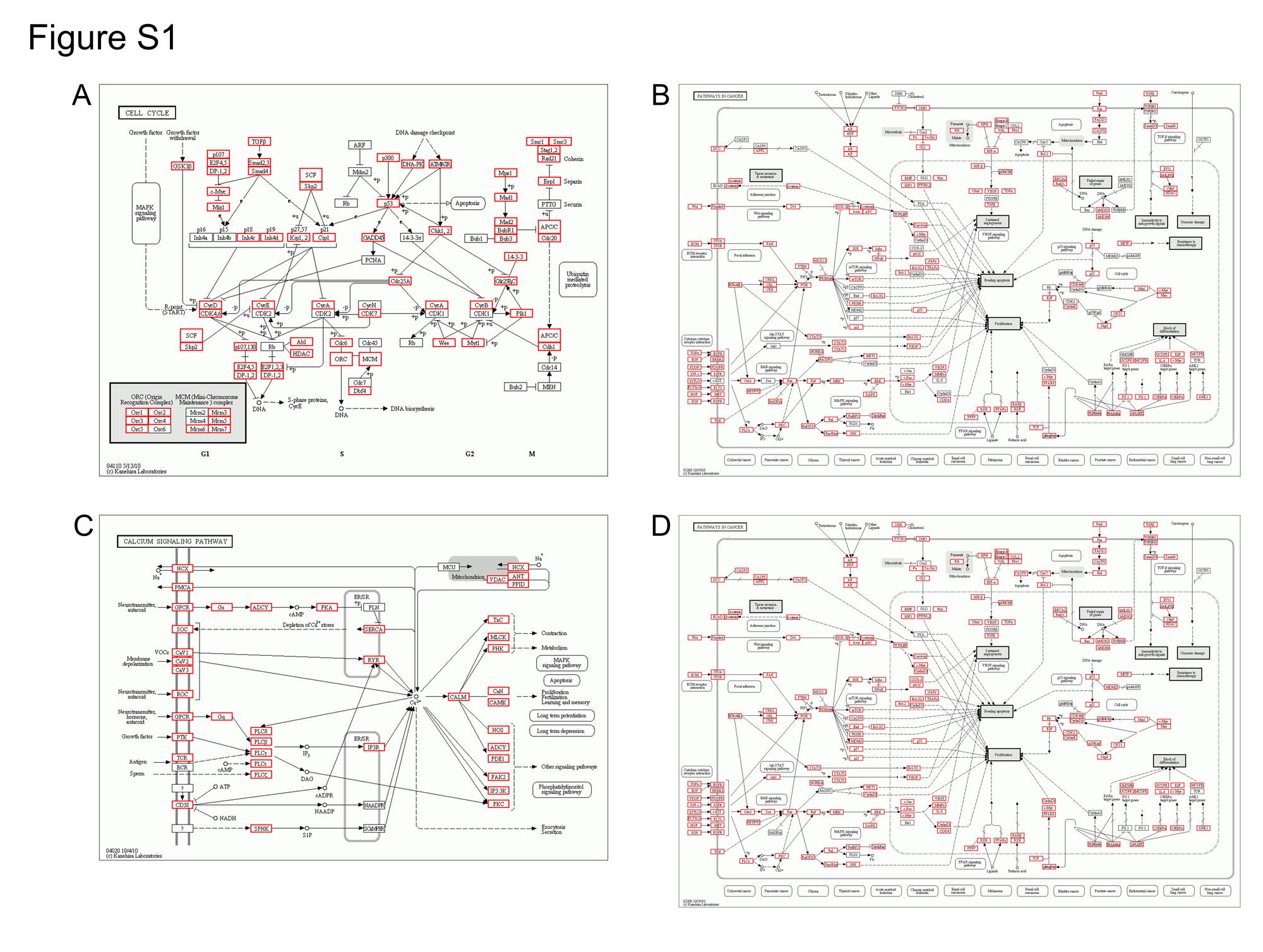

Supplement: Figure S1 — Genes and KEGG pathways that are predicted to be targeted by changed miRNAs. (A) Genes and cell cycle pathway targeted by up-regulated miRNAs. (B) Genes and pathways in cancer targeted by up-regulated miRNAs. (C) Genes and calcium signaling pathway targeted by up-regulated miRNAs. (D) Genes and pathways in cancer targeted by down-regulated miRNAs. Genes in red boxes indicate that are targeted by at least one miRNA. (TIF) [file pone.0071895.s001.tif]
